# Secondary Creep Analysis of FG Rotating Cylinder with Exponential, Linear and Quadratic Volume Reinforcement

**DOI:** 10.3390/ma15051803

**Published:** 2022-02-28

**Authors:** Manoj Sahni, Parth Dinesh Mehta, Ritu Sahni, Ernesto León-Castro, Luis F. Espinoza-Audelo

**Affiliations:** 1 Department of Mathematics, School of Technology, Pandit Deendayal Energy University, Gandhinagar 382426, Gujarat, India; mehtaparths@gmail.com (P.D.M.); ritusrivastava1981@gmail.com (R.S.); 2 Department of Management, Faculty of Economics and Administrative Sciences, Universidad Católica de la Santísima Concepción, Concepción 4030000, Chile; eleon@ucsc.cl; 3 Department of Industrial Engineering, Tecnológico Nacional de Mexico/Instituto Tecnológico de Culiacan, Culiacan 80225, Mexico; luis.ea@culiacan.tecnm.mx

**Keywords:** functionally graded cylinder, stress–strain, secondary creep, isotropic, internal/external pressure

## Abstract

Creep is an irreversible time-dependent deformation in which a material under constant mechanical stress and elevated temperature for a considerably prolonged period of time, starts to undergo permanent deformation. Creep deformation occurs in three stages namely, primary, secondary and tertiary. Out of these three stages, secondary or steady state creep is particularly an area of engineering interest as it has almost a constant creep rate. Creep deformation plays a significant role in understanding effective service life of an engineering component working under high temperature conditions as such components such as super-heater and re-heater tubes and headers in a boiler, jet engines operating at temperature as high as 1200 ${}^{\circ }$C, usually experience a failure or rupture due to creep phenomenon. Design engineers keep a close attention on working stress conditions and elevated temperature under which an engineering component is expected to work as these conditions determine the onset of creep behavior in an engineering component. By recognizing the parameters of material response to creep behavior, engineers can analyse the useful service life and hazardous working conditions for an engineering components. Recognizing the creep phenomenon as high temperature design limitation, ASME Boiler and Pressure Vessel Code have provided guidelines on maximum allowable stresses for materials to be used in creep range. One of the criteria for determination of allowable stresses is 1% creep deformation of material in 100,000 h of service. Thus, the study of creep behavior in engineering components pertaining to high stress and temperature working conditions is very important as it affects the reliability and performance of the engineering components. The aim of our study is to understand the behavior of secondary creep deformation so that an advanced reinforced functionally graded material with better creep resistance, can be designed. In this paper, a secondary creep analysis of functionally graded (FG) thick-walled rotating cylinder under internal and external pressure is conducted. The novelty of the model intends to specify secondary creep stresses and strains by employing exponential, linear and quadratic volume reinforcement for $SiC_{p}$ ceramic in Al metal matrix in radial direction. This will help us to understand the effect of volume reinforcement in FG cylinder under internal/external pressure and rotating centrifugal body force by obtaining secondary creep stresses and strains. The response of the FG cylinder with isotropic material is analyzed and the solution for stress–strain rates in radial and tangential directions are obtained in closed form. Comparison of steady state creep stresses and strains under exponential, linear and quadratic volume reinforcement profiles are discussed and presented graphically.

## 1. Introduction

Functionally Graded Materials (FGMs) are an advanced class of heterogeneous composite materials in which the composition and the structure vary continuously over the volume of the body. This smooth variation manifests itself in the form of continuous gradation of material, mechanical and thermal properties. There are many areas of applicability of functionally graded materials such as engineering, aerospace industry, energy sector, automobiles, defense, etc. FGMs were primarily designed for a space plane project in 1984, Japan, to provide mechanical strength and thermal resistance under very high surface temperature of 2000 K and a temperature gradient of 1000 K across a 10 mm section. Considering the effectivity of FGMs under such high temperature conditions, it is widely used in the tailoring of thick-walled cylinders, which are used in the structuring of pressure vessels for storing industrial gases and transportation of highly pressurized fluids, airplane fuselage, nuclear sector as light water reactors, steam generator tubes, gun barrel, submarine, vacuum chamber, etc. In these applications the thick-walled cylinder undergoes severe working conditions under high temperature, so it is important to understand how FGMs can protect the cylinders from deformations such as creep, which is caused due to high temperature and mechanical loading over the period of time. There are three stages of creep, namely, primary, secondary and tertiary. Out of these three, the secondary stage of creep is of prime importance as strain rates are near about constant over a period of time whereas in the primary as well as the tertiary stage, strain rates are very high and increases exponentially in the tertiary stage, leading to a fracture of the body. Many researchers have studied creep phenomena in functionally graded materials in order to understand how effectively creep stress–strain is handled. You et al. [1] studied steady state creep analysis on a thick-walled cylindrical pressure vessel made up of functionally graded material subjected to internal and external pressure using Norton’s law. Chen et al. [2] studied creep behavior of a functionally graded cylinder under both internal and external pressures by proposing an asymptotic method and presented numerical results for radial, tangential and axial stresses at different time steps. Sharma et al. [3] used Seth’s transition theory and obtained elastic–plastic stresses for a transversely isotropic thick-walled rotating cylinder under internal pressure and angular rotation. Atabakhshian and Loghman [4] studied time-dependent creep behavior of hollow circular rotating cylinders made up of exponentially graded material under the influence of internal pressure, temperature loading and centrifugal body force. Nejad et al. [5] derived exact closed-form solutions as well as finite element analysis for stresses and the displacements in pressurized thick spherical shells made of functionally graded materials with exponentially varying properties subjected to internal and external pressure. Sharma et al. [6] conducted safety analysis for thermal non-homogeneous thick-walled circular cylinder under internal and external pressure by using transition theory based on generalized principal Lebesgue strain measure in order to understand how the possibility of material fracture can be minimized. Sahni and Sharma [7] obtained creep stresses in a transversely isotropic thick-walled rotating circular cylinder using Seth’s transition theory and measured the effect of external pressure and rotation. Kashkoli and Nejad [8] presented an analytical solution for the time dependent creep analysis of an internally and externally pressurized, thick-walled cylindrical pressure vessel subjected to internal heat flux. Vedeld et al. [9] derived exact three dimensional, closed-form analytical solutions suitable in practical design contexts for uniformly heated, pressurized, two-layer elastic and isotropic cylinders. Loghman and Shayestemoghadam [10] obtained history of creep stresses and deformations of a nanocomposite rotating cylinder made of polypropylene reinforced by MWCNTs using Burgers viscoelastic creep model under magneto-thermo-mechanical loadings. Kalali et al. [11] provided an analytical solution for predicting stress components of a strain hardening cylinder based on the von-Mises yield criterion under plane–stress conditions by assuming an isotropic material model. Sharma and Yadav [12] analyzed thermal creep stresses in thick-walled cylinder with radially varying compressibility under the effect of internal and external pressure. Celebi et al. [13] presented a general solution for one-dimensional steady-state thermal and mechanical stresses in a hollow thick cylinder made of a functionally graded material. Bakhshizadeh et al. [14] solved the time-dependent problem of magneto-hygro-thermoelastic creep in an axisymmetric thick cylinder made of functionally graded material under the effect internal and external pressure. Sahni et al. [15] conducted secondary creep analysis in a rotating functionally graded cylinder with exponential volume reinforcement in the radial direction and observed the behavior of the FG cylinder in terms of creep stress–strain and under the effect of internal pressure as well as exponentially centrifugal force. Habib et al. [16] presented a mathematical analysis of stresses and strains of FG cylinder with exponential variation of material properties in the radial direction and under thermo-mechanical loading. Kashkoli et al. [17] using first-order shear deformation theory presented a theoretical solution for thermo-mechanical creep analysis in FG thick-walled cylindrical pressure vessel with variable thickness. Çallioğlu et al. [18] studied elastic–plastic regions and calculated stresses–strains in FG disk as well as cylinder with variable material properties under the influence of constant angular velocity and material gradient parameters. Influence of thermo-mechanical stresses in such a pressurized FG disk, tailored in power law form with radially varying material properties, namely, Young’s modulus, density and coefficient of thermal expansion were further investigated under linear and quadratic thermal loading conditions [19]. In order to reduce the magnitude of tangential stresses in thick-walled functionally graded cylinder, researchers have considered a sandwich composition of functionally graded material at an inner layer and composite material at an outer layer of the cylinder. The investigation revealed that material composition of a sandwich cylinder has significant influence on the behavior of radial and tangential stress under thermo-mechanical, magneto-thermo-mechanical loading conditions, internal heat generation and convective boundary conditions [20,21,22]. [PDM]With the advent of functionally graded materials, research work on steady state creep stresses and strains has gained impetus due to its ability to provide reduced stress concentration and smooth variation of reinforcement material volume from one surface to other surface of the body. A higher reinforcement of material and grain size provides better resistance to creep deformation in a material body. Rearchers have used various mathematical methods such as Runge Kutta 4th order, finite difference method (FDM), shooting method, complementary function method (CFM), homotopy analysis method for obtaining the solution of steady state creep in disks and cylinders made up of functionally graded materials. Creep models based on iterative technique, finite element method (FEM) and Seth’s transition theory for obtaining stress distribution in functionally graded rotating disks and cylinders can be also found in the literature [23,24,25]. Nie and Batra [26] studied the problem of material tailoring to obtain desired stress field in hollow cylinder made up of functionally graded in-compressible material with radially varying shear modulus. In this research work, we have considered exponential volume reinforcement of $SiC_{p}$ in radial direction of thick-walled isotropic rotating cylinder and the response of cylinder is analyzed. Further, comparison of creep stresses–strains under linear and quadratic reinforcement profiles for FG cylinder under internal/external pressure cases and volumetric centrifugal force is presented.

## 2. Secondary Creep Phenomenon in Engineering Applications

### 2.1. Problem Description of Functionally Graded Cylinder in Engineering

**Figure 1:**
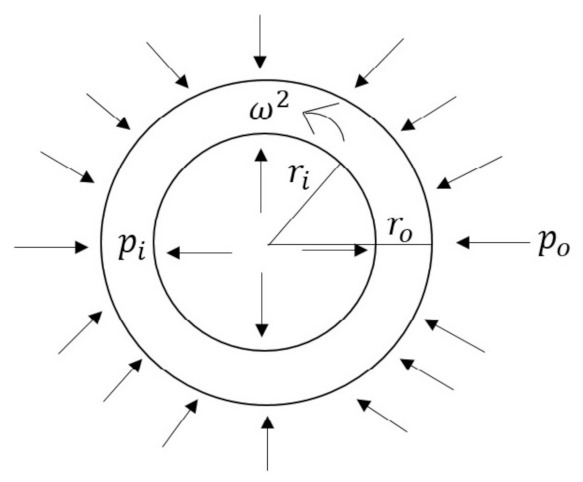
[PDM]Cross-section of pressurized functionally graded rotating cylinder.

As shown in Figure 1, we consider an axisymmetric thick-walled cylinder with inner radius $\left(r_{i}\right)$ and outer radius $\left(r_{o}\right)$, rotating with angular speed, $\omega^{2}$rad/s. The cylinder is subjected to compressive pressure at internal and external radius with $p_{i}$ and $p_{o}$, respectively. Pressurized circular cylinder structural design is widely used in the aerospace engineering field and energy sector for nuclear reactor power plants. Pressurized water reactors $\left(PWR\right)$ use circular cylinders with both, internal and external pressure and having isotropic material properties. In a steam generator tube, an external pressure can be used to assist the flow of primary coolant outside the cylindrical tube whereas internal pressure can assist the secondary water flow inside the cylindrical tube [25]. In such working conditions, material strength or safety factor, under pressure loads together with other surface and body forces, is to be critically analyzed to avoid the bursting or rupture of the pressurized cylindrical body [27]. Creep failures are characterized by bulging or blisters in the tube, intergranular voids and cracks in the micro-structure and thick-edged fractures often with very little obvious ductility. After an unfortunate incident involving a nuclear accident, in 2011, at the Fukushima Daiichi Nuclear Power Plant in Ōkuma, Fukushima, Japan, scientists around the world are working on establishing an advanced model for material creep response, prediction of creep damage and improving the accuracy of creep fracture position and time in reactor pressure vessels [28].

### 2.2. Mathematical Modeling for Secondary Creep Analysis of FG Cylinder

**Figure 2:**
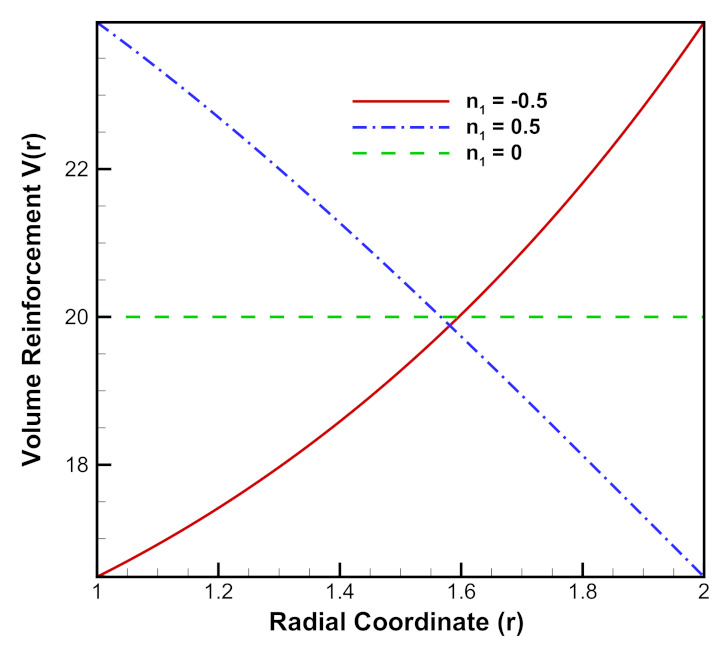
[PDM]Exponential volume reinforcement (vol%) of $SiC_{p}$ in Al metal matrix along radius *r* (in cm) of cylinder.

An axisymmetric thick-walled functionally graded (FG) cylinder with inner radii $r_{i}$ and outer radii $r_{o}$ as depicted in Figure 1, is made up of Al metal matrix reinforced with radially varying content of $SiC_{p}$ given as,

(1) $$V\left(r\right)=V_{o}\exp\left(-n_{1}{\left(\frac{r}{r_{o}}\right)}^{2}\right).$$

where $n_{1}$ is the reinforcement parameter through which the reinforcement of $SiC_{p}$ can be increased $\left(n_{1}<0\right)$ or decreased $\left(n_{1}>0\right)$ from inner to outer radii of the cylinder as shown in Figure 2.

The average $SiC_{p}$ content is given as,

(2) $$V_{avg}=-\frac{V_{o}\left(\exp\left(-n_{2}r_{o}^{2}\right)-\exp\left(-n_{2}r_{i}^{2}\right)\right)}{n_{2}\left(r_{o}^{2}-r_{i}^{2}\right)}$$

where, $n_{2}=\frac{n_{1}}{r_{o}^{2}}$. From the above relation (2), volume content at outer radii $V_{o}$ can be obtained as,

(3) $$V_{o}=-\left(\frac{V_{avg}\;n_{2}\left(r_{o}^{2}-r_{i}^{2}\right)}{\exp\left(-n_{2}\;r_{o}^{2}\right)-\exp\left(-n_{2}\;r_{i}^{2}\right)}\right)$$

The thick-walled functionally graded cylinder is subjected to internal/external pressure $p_{i}$ and $p_{o}$, respectively, which is kept constant during the loading history. A volumetric centrifugal body force, $\rho \left(r\right)\omega^{2}r$ is acting in radial direction with an angular velocity $\omega^{2}=50$ rad/s. The density $\rho \left(r\right)$ of functionally graded cylinder is given as,

(4) $$\rho \left(r\right)=\rho_{m}+0.01\left(\rho_{c}-\rho_{m}\right)\exp\left(-n_{1}{\left(\frac{r}{r_{o}}\right)}^{2}\right)$$

where, $\rho_{m}=2.7\;g/{\mathrm{cm}}^{3}$ is the density of Al metal and $\rho_{c}=3.21\;g/{\mathrm{cm}}^{3}$ is the density of $SiC_{p}$ ceramic. The functionally graded cylinder with cylindrical polar coordinates $\left(r,\theta ,z\right)$ is considered under plain strain in which the axial strain rate becomes zero due to a longer axial dimension as compared to radial and tangential dimensions. Thus, the axial strain rate, $\dot{\epsilon_{z}}=0$. Further, the material is assumed to be incompressible, due to which the sum of strain rates in radial, tangential and axial directions becomes zero, i.e., $\dot{\epsilon_{r}}+\dot{\epsilon_{\theta }}+\dot{\epsilon_{z}}=0$ [23,26] and the elastic deformations are neglected as compared to creep deformations.

The steady state creep in FG cylinder is analyzed using Norton’s law given as [1],

(5) $$\dot{\epsilon_{e}}=B{\sigma_{e}}^{n}$$

where, $\dot{\epsilon_{e}}$ is the effective strain rate, $\sigma_{e}$ is the effective stress, $B\left(r\right)$ and $n\left(r\right)$ are material parameters which depend on variable reinforcement in radial direction in FG cylinder and are given as,

(6) $$B\left(r\right)=B_{o}{\left[\frac{V\left(r\right)}{V_{avg}}\right]}^{n_{3}}\;\mathrm{and}\;n\left(r\right)=n_{o}{\left[\frac{V\left(r\right)}{V_{avg}}\right]}^{-n_{3}}.$$

Here $B_{0}$, $n_{0}$ are creep constants and $n_{3}$ is the gradation index. The radial and tangential strain rates in the cylinder are given by [5],

(7) $$\dot{\epsilon_{r}}=\frac{d{\dot{u}}_{r}}{dr}\;\mathrm{and}\;\dot{\epsilon_{\theta }}=\frac{{\dot{u}}_{r}}{r}$$

where, $\dot{\epsilon_{r}}$, $\dot{\epsilon_{\theta }}$, $\dot{u}$ are rates of radial strain, tangential strain and displacement, respectively. The compatibility condition are obtained as [15],

(8) $$\dot{\epsilon_{r}}=\dot{\epsilon_{\theta }}+r\frac{d\dot{\epsilon_{\theta }}}{dr}$$

The constitutive relations for multiaxial creep phenomena are given as [15],

(9) $$\begin{array}{cc} \; & \dot{\epsilon_{r}}=\frac{\dot{\epsilon_{e}}}{\left(G+H\right)\sigma_{e}}\left[G\left(\sigma_{r}-\sigma_{z}\right)+H\left(\sigma_{r}-\sigma_{\theta }\right)\right]\\ \; & \dot{\epsilon_{\theta }}=\frac{\dot{\epsilon_{e}}}{\left(G+H\right)\sigma_{e}}\left[F\left(\sigma_{\theta }-\sigma_{z}\right)+H\left(\sigma_{\theta }-\sigma_{r}\right)\right]\\ \; & \dot{\epsilon_{z}}=\frac{\dot{\epsilon_{e}}}{\left(G+H\right)\sigma_{e}}\left[F\left(\sigma_{z}-\sigma_{\theta }\right)+G\left(\sigma_{z}-\sigma_{r}\right)\right] \end{array}$$

where *F*, *G* and *H* are anisotropic constants, $\dot{\epsilon_{e}}$ and $\sigma_{e}$ are effective strain rate and effective stress, respectively. The effective stress in functionally graded cylinder is estimated according to Hill’s yield criterion given as [15],

(10) $$\sigma_{e}={\left(\frac{1}{\left(G+H\right)}F{\left(\sigma_{\theta }-\sigma_{z}\right)}^{2}+G{\left(\sigma_{z}-\sigma_{r}\right)}^{2}+H{\left(\sigma_{r}-\sigma_{\theta }\right)}^{2}\right)}^{\frac{1}{2}}$$

The governing differential equation for a rotating FG cylinder under rotation is given by [15],

(11) $$r\frac{d\sigma_{r}}{dr}+\sigma_{r}-\sigma_{\theta }+\rho \left(r\right)\omega^{2}r^{2}=0$$

where $\sigma_{r}$, $\sigma_{\theta }$, ρ and ω are stresses—radial and tangential, density and angular speed, respectively. Further, the internal as well as external pressure applied are kept constant during the loading and hence boundary conditions are given as,

$$\sigma_{r}=-p_{i}\;\mathrm{at}\;r=r_{i}\;\mathrm{and}\;\sigma_{r}=-p_{o}\;\mathrm{at}\;r=r_{o}$$

where, the negative sign indicates the compressive nature of radial stress.

Since, $\dot{\epsilon_{r}}+\dot{\epsilon_{\theta }}+\dot{\epsilon_{z}}=0$ and from plain strain condition $\left(\dot{\epsilon_{z}}=0\right)$, Equation (7) gives,

(12) $${\dot{u}}_{r}=\frac{C}{r}$$

where, *C* is constant of integration. From Equations (12) and (7), strain rates are obtained as,

(13) $${\dot{\epsilon }}_{r}=-\frac{C}{r^{2}}\;\mathrm{and}\;{\dot{\epsilon }}_{\theta }=\frac{C}{r^{2}}$$

Using plain strain condition and taking $\dot{\epsilon_{z}}=0$, the third equation from set of Equations (9) can be written as,

(14) $$\sigma_{z}=\frac{G\sigma_{r}+F\sigma_{\theta }}{F+G}$$

Using the expression (14) for axial stress $\sigma_{z}$ in Equation (10), we obtain

(15) $$\sigma_{e}=\frac{\sigma_{\theta }-\sigma_{r}}{\sqrt{\left(H+G\right)}}{\left(\frac{FG+GH+HF}{F+G}\right)}^{\frac{1}{2}}$$

Substituting Equations (13) and (14) in the first equation from set of Equations (9), we have

(16) $$\sigma_{\theta }-\sigma_{r}=\frac{\left(F+G\right)\left(H+G\right)\sigma_{e}C}{\left(FG+GH+HF\right)\dot{\epsilon_{e}}r^{2}}$$

Using Equations (5) and (15) in (16) we obtain,

(17) $$\sigma_{\theta }-\sigma_{r}=\frac{I_{1}}{r^{\frac{2}{n}}}$$

where

$$I_{1}={\left(\frac{\left(F+G\right)\left(H+G\right)}{\left(FG+GH+HF\right)}\right)}^{\frac{n+1}{2n}}\frac{C^{\frac{1}{n}}}{B^{\frac{1}{n}}}$$

Substituting the above Equation (17) in the governing differential Equation (11) for a rotating FG cylinder and integrating from $r_{i}$ to *r*, we obtain (Appendix A)

(18) $$\sigma_{r}=\int_{r_{i}}^{r}\frac{I_{1}}{r^{\frac{n+2}{n}}}dr-\frac{\rho_{m}\omega^{2}\left(r^{2}-{r_{i}}^{2}\right)}{2}+\frac{\left(\rho_{c}-\rho_{m}\right)\omega^{2}}{100}\frac{{r_{o}}^{2}}{2n_{1}}\left[e^{-n_{1}\left(\frac{r^{2}}{{r_{o}}^{2}}\right)}-e^{-n_{1}\left(\frac{{r_{i}}^{2}}{{r_{o}}^{2}}\right)}\right]-p_{i}$$

Substituting above Equation (18) in (17), we obtain

(19) $$\sigma_{\theta }=\frac{I_{1}}{r^{\frac{2}{n}}}+\sigma_{r}$$

The integration constant *C* is obtained using the boundary condition at $r=r_{o}$

(20) $$C={\left(\frac{\left(r_{o}^{2}-r_{i}^{2}\right)\frac{\rho_{m}\omega^{2}}{2}+p_{i}-p_{o}-\frac{0.01\left(\rho_{c}-\rho_{m}\right)\omega^{2}{r_{o}}^{2}}{2n_{1}}\left(e^{-n_{1}}-e^{-n_{1}{\left(\frac{r_{i}}{r_{o}}\right)}^{2}}\right)}{\int_{r_{i}}^{r_{o}}{\left(\frac{\left(F+G\right)\left(H+G\right)}{\left(FG+GH+HF\right)}\right)}^{\frac{n+1}{2n}}\frac{1}{r^{\frac{n+2}{n}}B^{\frac{1}{n}}}dr}\right)}^{n}$$

Substituting Equations (14) and (15) into first and second equations from the set of Equations (9), we obtain

(21) $$\dot{\epsilon_{\theta }}=-\dot{\epsilon_{r}}=\frac{\dot{\epsilon_{e}}}{\sqrt{\frac{\left(F+G\right)\left(H+G\right)}{\left(FG+GH+HF\right)}}}$$

## 3. Results and Discussion

The values of creep parameters [2,24] $B_{o}$, $n_{o}$ and $n_{3}$ are taken as, $B_{o}=2.77\times 10^{-6}$, $n_{o}=3.75$ and $n_{3}=0.7$, respectively. Here, for an isotropic cylinder, F=G=H. The average volume content for $SiC_{p}$ is considered as $V_{avg}=20\%$. The impact of exponential, linear and quadratic volume reinforcement on creep stresses (in MPa)—strains in rotating functionally graded cylinder under internal pressure is discussed. The value of creep stress is obtained in MPa unit and plotted over radial coordinate *r* in cm unit.

### 3.1. Effect of Exponential Volume Reinforcement

**Figure 3:**
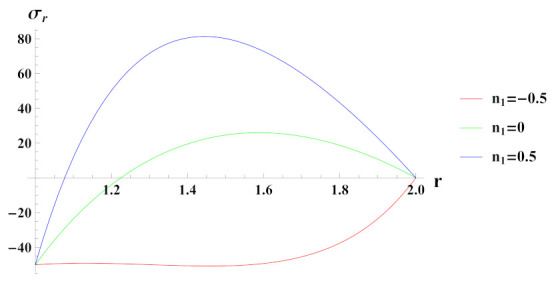
[PDM]Radial stress (in MPa) along the radius *r* (in cm) of the cylinder, under the effect of rotation ($\omega^{2}$ = 50 rad/s), internal pressure $p_{i}=50\;\mathrm{MPa}$ and exponential volume reinforcement.

Figure 3 depicts radial stresses in a rotating cylinder with an exponential volume reinforcement of $SiC_{p}$ in Al metal matrix and under internal pressure $p_{i}$. It can be seen that when cylinder is under the effect of internal pressure of 50 MPa and with decreasing exponential volume reinforcement profile $n_{1}=0.5$, radial stress is compressive at inner radial points and becomes tensile as it moves towards the outer radius. Under increasing exponential volume reinforcement $\left(n_{1}=-0.5\right)$ from inner radius of the cylinder to its outer radius, radial stress is compressive throughout the radius of cylinder and decreases towards the outer radius. When volume of reinforcement is kept constant along the radius of cylinder, i.e., $n_{1}=0$, radial stress is compressive at inner radial points and becomes compressive along the outer radial points of the cylinder. The magnitude of radial stress in this case is significantly lower as compared to decreasing volume reinforcement profile.

**Figure 4:**
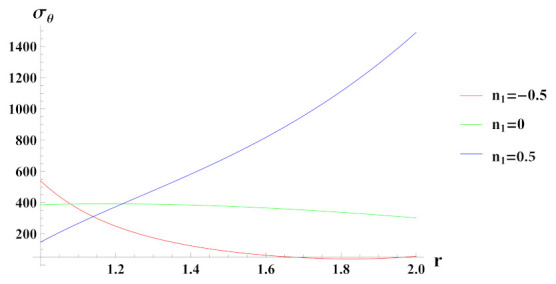
[PDM]Tangential stress (in MPa) along the radius *r* (in cm) of the cylinder, under the effect of rotation ($\omega^{2}$ = 50 rad/s), internal pressure $p_{i}=50\;\mathrm{MPa}$ and exponential volume reinforcement.

Figure 4 presents tangential stresses in a rotating cylinder with an exponential volume reinforcement and an internal pressure of 50 MPa. It can be seen that under decreasing volume reinforcement profile, $n_{1}=0.5$, tangential stress increases towards the outer radius of the cylinder whereas in case of $n_{1}=0$ and $n_{1}=-0.5$, it is decreasing over the radius of cylinder. Tangential stress under increasing reinforcement profile, $n_{1}=-0.5$ is tensile except at some outer radial points.

**Figure 5:**
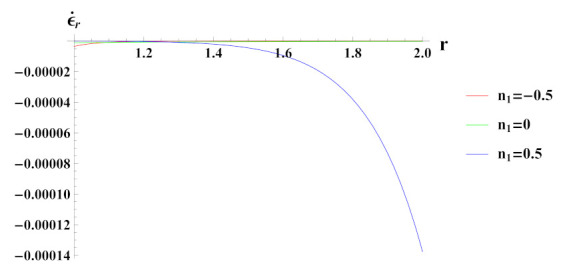
[PDM]Radial strain rate along the radius *r* (in cm) of the cylinder, under the effect of rotation ($\omega^{2}$ = 50 rad/s), internal pressure $p_{i}=50\;\mathrm{MPa}$ and exponential volume reinforcement.

As observed in Figure 5, radial strain rate under internal pressure is compressive in nature. The radial strain rate is highly compressive in cylinder with reinforcement $n_{1}=0.5$ but decreases in magnitude for $n_{1}=0.5$ and $n_{1}=0$.

**Figure 6:**
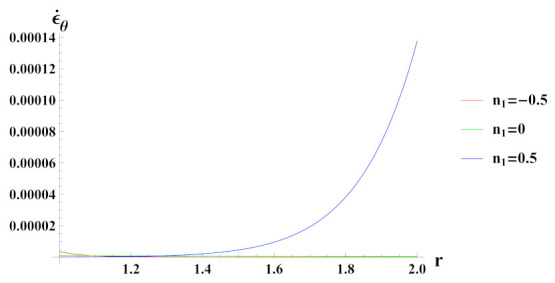
[PDM]Tangential strain rate along the radius *r* (in cm) of the cylinder, under the effect of rotation ($\omega^{2}$ = 50 rad/s), internal pressure $p_{i}$ = 50 MPa and exponential volume reinforcement.

Figure 6 presents tangential strain rate for a cylinder under internal pressure and rotation. It is seen that the tangential strain rate is positive for all the cases of reinforcement. In a cylinder with decreasing content of reinforcement ($n_{1}=0.5$) of $SiC_{p}$, tangential strain rate increases at the outer radius, exponentially.

**Figure 7:**
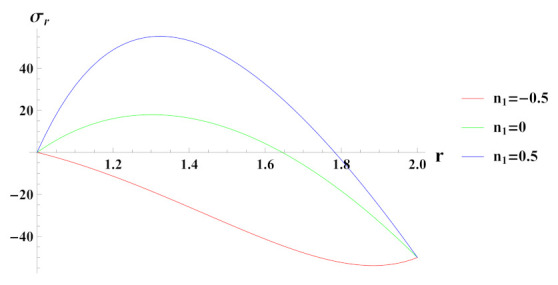
[PDM]Radial stress (in MPa) along the radius *r* (in cm) of the cylinder, under the effect of rotation ($\omega^{2}$ = 50 rad/s), external pressure $p_{o}$ = 50 MPa and exponential volume reinforcement.

Figure 7 depicts radial stresses in a rotating cylinder with an exponential volume reinforcement under the effect of external pressure. It can be observed that under increasing reinforcement $n_{1}=-0.5$, radial stress is compressive in nature throughout the radius and starts to decrease in magnitude at outer radial points due to imposed boundary pressure conditions. For $n_{1}=0$ and $n_{1}=0.5$ cases, radial stress is tensile but becomes compressive towards the outer radius. Here, it can be noted that the compressiveness of radial stress under $n_{1}=-0.5$ reinforcement case is high towards the outer radius in comparison to reinforcement case $n_{1}=0.5$. This indicates the increase in strength of the material of cylinder.

**Figure 8:**
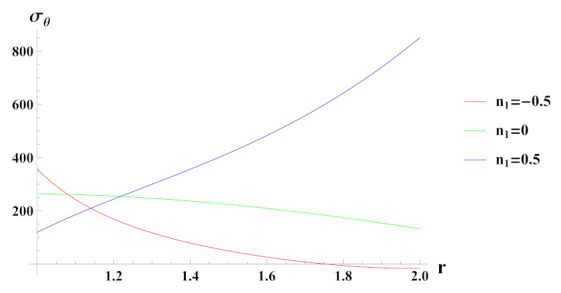
[PDM]Tangential stress (in MPa) along the radius *r* (in cm) of the cylinder, under the effect of rotation ($\omega^{2}$ = 50 rad/s), external pressure $p_{o}$ = 50 MPa and exponential volume reinforcement.

Figure 8 presents tangential stresses in a rotating FG cylinder in presence of external pressure. It can be seen that tangential stress is tensile in nature except towards the outer radius for $n_{1}=-0.5$ reinforcement case. It is evident from the graph that tangential stress towards outer radius becomes exceedingly tensile due to decreasing reinforcement of $SiC_{p}$ at outer radial points of the cylinder.

**Figure 9:**
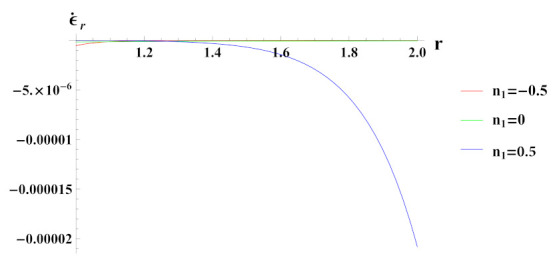
[PDM]Radial strain rate along the radius *r* (in cm) of the cylinder, under the effect of rotation ($\omega^{2}$ = 50 rad/s), external pressure $p_{o}$ = 50 MPa and exponential volume reinforcement.

Radial strain rate under decreasing reinforcement profile $n_{1}=0.5$ as observed from Figure 9, is compressive and it increases exponentially towards outer radial points. Radial strain rate under increasing reinforcement profile case $n_{1}=-0.5$, is higher in magnitude at inner radial points but decreases towards the outer radius, whereas the exact opposite behavior is observed for the $n_{1}=0$ case.

**Figure 10:**
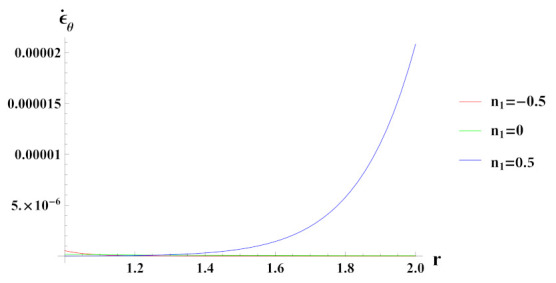
[PDM]Tangential strain rate along the radius *r* (in cm) of the cylinder, under the effect of rotation ($\omega^{2}$ = 50 rad/s), external pressure $p_{o}$ = 50 MPa and exponential volume reinforcement.

Tangential strain rate under decreasing reinforcement profile from inner to outer radius ($n_{1}=0.5$) as seen in Figure 10, is tensile and increases exponentially along the radius of the cylinder. It is noted that tangential strain rate under increasing reinforcement case $n_{1}=-0.5$ is higher in magnitude at inner radial points and decrease at the outer radial of the cylinder, whereas the opposite behavior in case $n_{1}=0$ reinforcement case is observed. In reinforcement cases $n_{1}=0$ and $n_{1}=-0.5$, tangential strain rate is again tensile and ceases towards the outer radial points.

### 3.2. Effect of Linear Volume Reinforcement

The volume reinforcement of $SiC_{p}$ ceramic in Al metal matrix is given as,

(22) $$V\left(r\right)=V_{o}\left(1+mr\right)$$

where, *m* is material reinforcement gradation index. The volume content at outer radii $V_{o}$ can be obtained as,

(23) $$V_{o}=\frac{V_{avg}\left(r_{o}^{2}-r_{i}^{2}\right)}{\left(r_{o}^{2}-r_{i}^{2}\right)+\frac{2}{3}m\left(r_{o}^{3}-r_{i}^{3}\right)}$$

**Figure 11:**
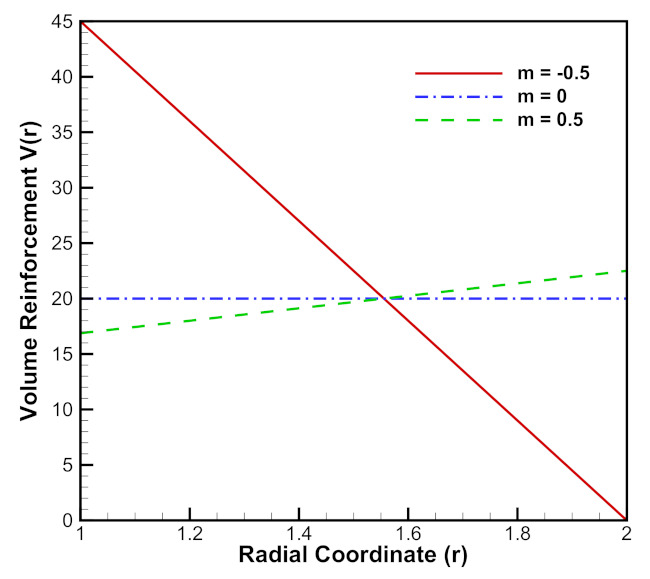
[PDM]Linear volume reinforcement (vol %) of $SiC_{p}$ in Al metal matrix along the radius *r* (in cm) of the cylinder.

Figure 11 depicts linear volume reinforcement of $SiC_{p}$ in Al metal matrix. When material reinforcement gradation index, m=0.5, volume reinforcement of $SiC_{p}$ increases from the inner radius of the cylinder to its outer radius and when m=−0.5, the opposite reinforcement behavior occurs, i.e., it decreases from the inner radius of the cylinder to its outer radius. For, m=0, the volume of $SiC_{p}$ remains constant for the entire radius of the cylinder.

Radial stress $\sigma_{r}$ in rotating cylinder under linear volume reinforcement profile is given as,

(24) $$\begin{array}{cc} \sigma_{r}= & \int_{r_{i}}^{r}\frac{I_{1}}{r^{\frac{n+2}{n}}}dr-p_{i}-\frac{\rho_{m}\omega^{2}\left(r^{2}-r_{i}^{2}\right)}{2}\\ \; & -\left(0.01\right)\left(\rho_{c}-\rho_{m}\right)\omega^{2}\left(\frac{\left(r^{2}-r_{i}^{2}\right)}{2}+\frac{m\left(r^{3}-r_{i}^{3}\right)}{3}\right) \end{array}$$

where,

$$I_{1}={\left(\frac{\left(F+G\right)\left(H+G\right)}{\left(FG+GH+HF\right)}\right)}^{\frac{n+1}{2n}}\frac{C^{\frac{1}{n}}}{B^{\frac{1}{n}}}$$

Here, $B\left(r\right)$ and $n\left(r\right)$ are given by Equation (6). The constant *C* is evaluated by using the boundary condition at $r=r_{o}$ and is given as,

(25) $$C={\left(\frac{-p_{o}+p_{i}+\frac{\rho_{m}\omega^{2}\left(r_{o}^{2}-r_{i}^{2}\right)}{2}+\left(0.01\right)\left(\rho_{c}-\rho_{m}\right)\omega^{2}\left(\frac{1}{2}\left(r_{o}^{2}-r_{i}^{2}\right)+\frac{m}{3}\left(r_{o}^{3}-r_{i}^{3}\right)\right)}{\int_{r_{i}}^{r_{o}}{\left(\frac{\left(F+G\right)\left(H+G\right)}{\left(FG+GH+HF\right)}\right)}^{\frac{n+1}{2n}}\frac{1}{r^{\frac{n+2}{n}}B^{\frac{1}{n}}}dr}\right)}^{n}$$

From Equations (14) and (17) axial stress $\sigma_{z}$ and tangential stress $\sigma_{\theta }$, respectively, in a rotating cylinder with linear volume reinforcement can be obtained. The radial and tangential strain rates in a rotating cylinder under linear volume reinforcement can be obtained from Equations (13) and (25).

**Figure 12:**
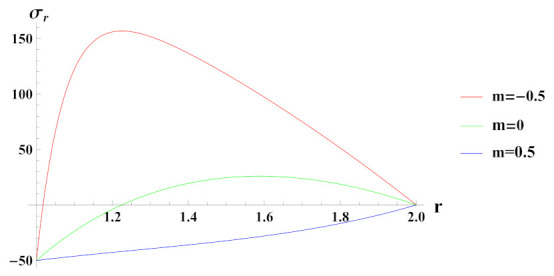
[PDM]Radial stress (in MPa) along the radius *r* (in cm) of the cylinder, under the effect of rotation ($\omega^{2}$ = 50 rad/s), internal pressure $p_{i}=50\;\mathrm{MPa}$ and linear volume reinforcement.

Figure 12 presents radial stress in cylinder under rotation and internal pressure. The reinforcement of $SiC_{p}$ in Al is increasing when m=0.5 and decreasing when m=−0.5 whereas for m=0 it is constant. It can be observed that under linearly increasing reinforcement, radial stress is compressive throughout the radius of cylinder with decreasing magnitude towards the outer radius whereas under decreasing reinforcement and composite profile, it is compressive at inner radial points due to the boundary condition but becomes tensile as we move along the radius of the cylinder.

**Figure 13:**
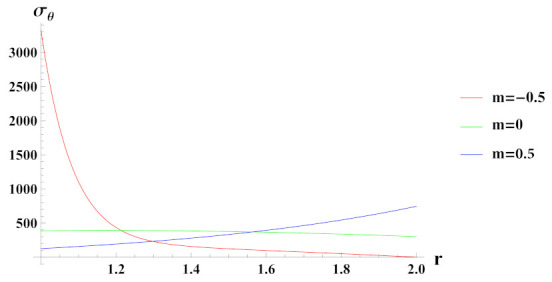
[PDM]Tangential stress (in MPa) along the radius *r* (in cm) of the cylinder, under the effect of rotation ($\omega^{2}$ = 50 rad/s), internal pressure $p_{i}=50\;\mathrm{MPa}$ and linear volume reinforcement.

From Figure 13, it can be seen that tangential stress under decreasing linear reinforcement profile is higher at internal radial points but decreases exponentially towards outer radial points of the cylinder. With increase in the content of $SiC_{p}$ over the radius of cylinder, tangential stress also increases from inner to outer radius with significantly lower magnitude as compared to decreasing reinforcement profile. When the material is graded as a composite, tangential stress decreases from inner to outer radius of the cylinder.

**Figure 14:**
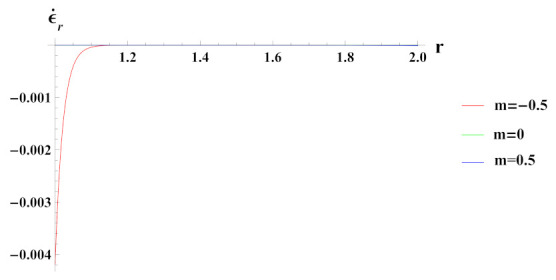
[PDM]Radial strain rate along the radius *r* (in cm) of the cylinder, under the effect of rotation ($\omega^{2}$ = 50 rad/s), internal pressure $p_{i}=50\;\mathrm{MPa}$ and linear volume reinforcement.

Radial strain rate as seen in Figure 14 is compressive at internal radial points and tensile at outer radial points of the cylinder for decreasing reinforcement profile. Under increasing reinforcement profile, strain rate in radial direction is higher at internal radial points but decreases at the outer radius. It increases from the inner radius of the cylinder to its outer radius under composite material gradation m=0 with compressiveness at inner radial points.

**Figure 15:**
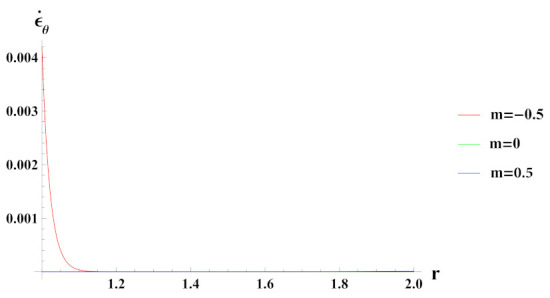
[PDM]Tangential strain rate along the radius *r* (in cm) of the cylinder, under the effect of rotation ($\omega^{2}$ = 50 rad/s), internal pressure $p_{i}=50\;\mathrm{MPa}$ and linear volume reinforcement.

Tangential strain rate under decreasing reinforcement profile m=−0.5, as observed from Figure 15 is tensile at internal radial points and becomes compressive at outer radial points of the cylinder. Tangential strain increases with increase in reinforcement of $SiC_{p}$ (m=0.5), throughout radii of the cylinder. In cylinder with composite material, tangential strain rate is tensile at inner radius but it becomes compressive towards the outer radius.

**Figure 16:**
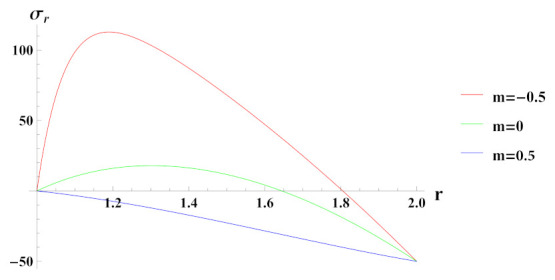
[PDM]Radial stress (in MPa) along the radius *r* (in cm) of the cylinder, under the effect of rotation ($\omega^{2}$ = 50 rad/s), external pressure $p_{o}$ = 50 MPa and linear volume reinforcement.

Figure 16 presents radial stress under the effect of external pressure in a rotating cylinder. Radial stress in a cylinder with increasing reinforcement profile is compressive in nature and its magnitude increases as we move along the radius of the cylinder. Under decreasing reinforcement profile, radial stress at internal radial points is tensile and decreases towards the outer radial points, becoming compressive in nature. Radial stress under composite material gradation, behaves similar to profile with decreasing reinforcement but has lower order of magnitude.

**Figure 17:**
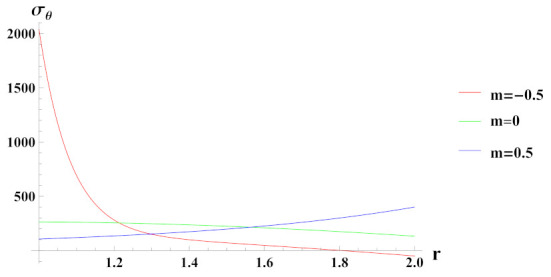
[PDM]Tangential stress (in MPa) along the radius *r* (in cm) of the cylinder, under the effect of rotation ($\omega^{2}$ = 50 rad/s), external pressure $p_{o}$ = 50 MPa and linear volume reinforcement.

As observed from Figure 17, tangential stress under the effect of external pressure in a rotating cylinder and under linearly increasing profile is lowest at inner radius, thereby, increasing towards the outer radius. Under decreasing reinforcement m=−0.5, tangential stress is high at inner radius but along the radius of cylinder it starts to decrease and becomes compressive towards the outer radius of the cylinder. Further, it can be seen that tangential stress in composite cylinder is tensile throughout the radius and decreases towards the outer radius.

**Figure 18:**
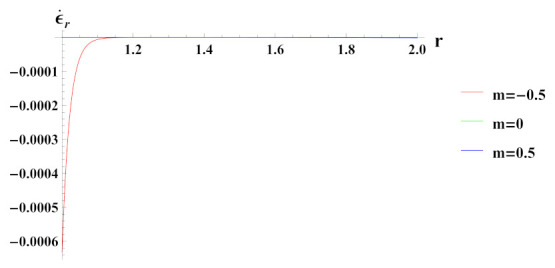
[PDM]Radial strain rate along the radius *r* (in cm) of the cylinder, under the effect of rotation ($\omega^{2}$ = 50 rad/s), external pressure $p_{o}$ = 50 MPa and linear volume reinforcement.

Figure 18 presents radial strain rate in externally pressurized cylinder under rotation. Radial strain rate for decreasing reinforcement profile m=−0.5, is highly compressive at inner radius and becomes tensile for longer part of the radius of the cylinder. Under increasing reinforcement profile m=0.5, radial strain rate is lower in magnitude as compared to m=−0.5 case but increases at outer radial points. In composite cylinder, radial strain rate is compressive at inner radius but as we go along the radius of the cylinder it becomes tensile in nature.

**Figure 19:**
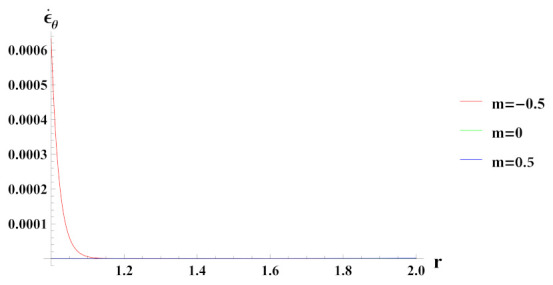
[PDM]Tangential strain rate along the radius *r* (in cm) of the cylinder, under the effect of rotation ($\omega^{2}$ = 50 rad/s), external pressure $p_{o}$ = 50 MPa and linear volume reinforcement.

Tangential strain rate in externally pressurized cylinder under rotation is shown in Figure 19. It can be seen that under linearly decreasing reinforcement profile m=−0.5, tangential strain rate at inner radial portion of the cylinder is high and starts to decrease with compressiveness along the outer radial points of the cylinder. Under increasing reinforcement profile m=−0.5, the tangential strain rate at the outer radial portion of the cylinder increases. In the composite cylinder, it is found to be higher at the inner radius as compared to cylinder with increasing reinforcement profile.

### 3.3. Effect of Quadratic Volume Reinforcement

The volume reinforcement of $SiC_{p}$ in Al metal matrix is given as,

(26) $$V\left(r\right)=V_{o}\left(1+m\left(r+r^{2}\right)\right)$$

where, *m* is material reinforcement gradation index. The volume content at outer radii $V_{o}$ can be obtained as,

(27) $$V_{o}=\frac{V_{avg}\left(r_{o}^{2}-r_{i}^{2}\right)}{\left(r_{o}^{2}-r_{i}^{2}\right)+\frac{2}{3}m\left(r_{o}^{3}-r_{i}^{3}\right)+\frac{m}{2}\left(r_{o}^{4}-r_{i}^{4}\right)}$$

**Figure 20:**
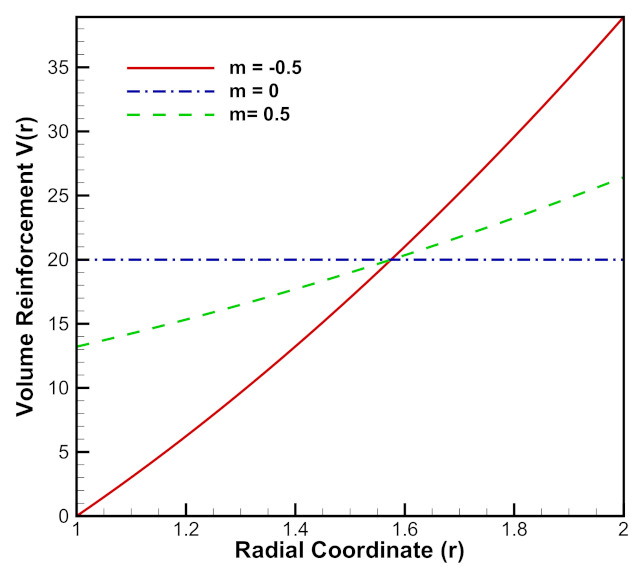
[PDM]Quadratic volume reinforcement (vol %) of $SiC_{p}$ in Al metal matrix along the radius *r* (in cm) of the cylinder.

Figure 20 depicts quadratically increasing volume reinforcement of $SiC_{p}$ ceramic in Al metal matrix along the radius of cylinder. Here, *m* is the volume reinforcement gradation. Volume reinforcement of $SiC_{p}$ increases from inner radius of the cylinder to its outer radius for different values of gradation, i.e., m=−0.5 and m=0.5. When m=0 the volume reinforcement of $SiC_{p}$ remains constant throughout the cylinder radii.

Radial stress $\sigma_{r}$ in cylinder under quadratic volume reinforcement profile and rotation can be given as,

(28) $$\begin{array}{cc} \sigma_{r}= & \int_{r_{i}}^{r}\frac{I_{1}}{r^{\frac{n+2}{n}}}dr-p_{i}-\frac{\rho_{m}\omega^{2}\left(r^{2}-r_{i}^{2}\right)}{2}\\ \; & -\left(0.01\right)\left(\rho_{c}-\rho_{m}\right)\omega^{2}\left(\frac{\left(r^{2}-r_{i}^{2}\right)}{2}+\frac{m\left(r^{3}-r_{i}^{3}\right)}{3}+\frac{m\left(r^{4}-r_{i}^{4}\right)}{4}\right) \end{array}$$

where,

$$I_{1}={\left(\frac{\left(F+G\right)\left(H+G\right)}{\left(FG+GH+HF\right)}\right)}^{\frac{n+1}{2n}}\frac{C^{\frac{1}{n}}}{B^{\frac{1}{n}}}$$

Here, $B\left(r\right)$ and $n\left(r\right)$ are given by Equation (6). The constant *C* is evaluated by using the boundary condition at $r=r_{o}$ and is given as,

(29) $$C={\left(\frac{-p_{o}+p_{i}+\frac{\rho_{m}\omega^{2}\left(r_{o}^{2}-r_{i}^{2}\right)}{2}+\frac{\left(\rho_{c}-\rho_{m}\right)\omega^{2}}{100}\left(\frac{\left(r_{o}^{2}-r_{i}^{2}\right)}{2}+\frac{m\left(r_{o}^{3}-r_{i}^{3}\right)}{3}+\frac{m\left(r_{o}^{4}-r_{i}^{4}\right)}{4}\right)}{\int_{r_{i}}^{r_{o}}{\left(\frac{\left(F+G\right)\left(H+G\right)}{\left(FG+GH+HF\right)}\right)}^{\frac{n+1}{2n}}\frac{1}{r^{\frac{n+2}{n}}B^{\frac{1}{n}}}dr}\right)}^{n}$$

For rotating cylinder with quadratic volume reinforcement, the expression for axial stress $\sigma_{z}$ and tangential stress $\sigma_{\theta }$ can be obtained using Equations (14) and (17), respectively. Further, radial and tangential strain rates can be obtained from Equations (13) and (25).

**Figure 21:**
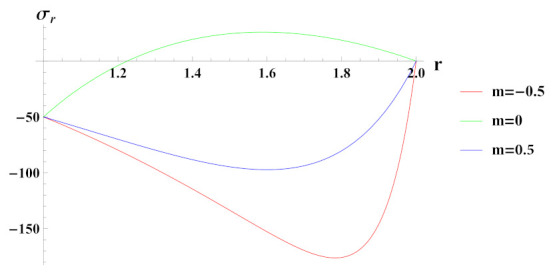
[PDM]Radial stress (in MPa) along the radius *r* (in cm) of the cylinder, under the effect of rotation ($\omega^{2}$ = 50 rad/s), internal pressure $p_{i}=50\;\mathrm{MPa}$ and quadratic volume reinforcement.

Figure 21 presents radial stress in a rotating cylinder under an internal pressure. It can be seen that radial stress in a cylinder with volume reinforcement m=−0.5 is highly compressive at outer radial points. Its magnitude increases from inner to outer radial points and starts to decreases along the outer radius due to imposed boundary condition. When the cylinder is under volume reinforcement profile, m=0.5, compressive nature of radial stress decreases as compared to decreasing reinforcement profile, m=−0.5. With constant gradation of cylinder, radial stress is compressive at inner radius due to imposed boundary condition and becomes tensile in nature along the radius of cylinder.

**Figure 22:**
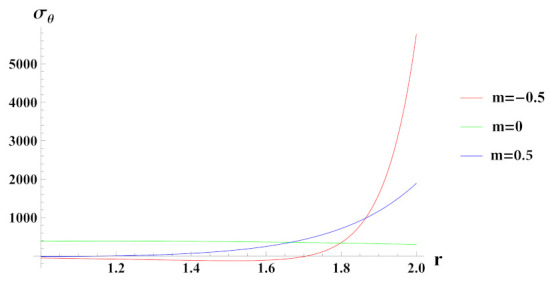
[PDM]Tangential stress (in MPa) along the radius *r* (in cm) of the cylinder, under the effect of rotation ($\omega^{2}$ = 50 rad/s), internal pressure $p_{i}=50\;\mathrm{MPa}$ and quadratic volume reinforcement.

From Figure 22, it can be seen that tangential stress in a rotating cylinder with volume reinforcement profile m=0.5 and under internal pressure, is tensile at all radial points of the cylinder. It increases from internal to external radial portion of the cylinder whereas under volume reinforcement case m=−0.5, tangential stress is compressive at inner radial points and becomes tensile with high magnitude at outer radial points of the cylinder.

**Figure 23:**
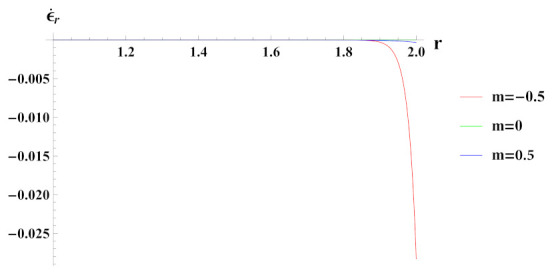
[PDM]Radial strain rate along the radius *r* (in cm) of the cylinder, under the effect of rotation ($\omega^{2}$ = 50 rad/s), internal pressure $p_{i}=50\;\mathrm{MPa}$ and quadratic volume reinforcement.

Compressive radial strain rate in an internally pressurized cylinder under rotation is depicted in Figure 23. It is highly compressive at outer radial points and decreases in magnitude at outer radius for m=0 and m=0.5 gradation cases.

**Figure 24:**
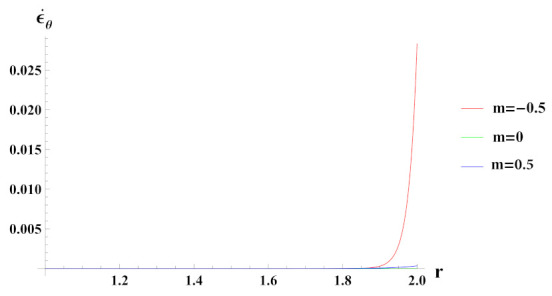
[PDM]Tangential strain rate along the radius *r* (in cm) of the cylinder, under the effect of rotation ($\omega^{2}$ = 50 rad/s), internal pressure $p_{i}=50\;\mathrm{MPa}$ and quadratic volume reinforcement.

Tangential strain rate as seen in Figure 24, in a rotating cylinder under internal pressure is tensile. When m=−0.5, it increases exponentially at outer radius whereas under m=0.5 case, it has lower magnitude throughout the radius of cylinder. It can be noted that under constant gradation profile, tangential strain rate is lowest at outer radial points.

**Figure 25:**
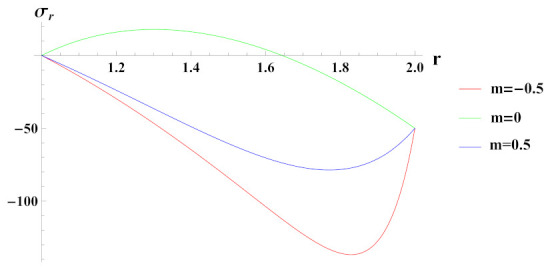
[PDM]Radial stress (in MPa) along the radius *r* (in cm) of the cylinder, under the effect of rotation ($\omega^{2}$ = 50 rad/s), external pressure $p_{o}$ = 50 MPa and quadratic volume reinforcement.

Figure 25 depicts radial stress in a rotating cylinder under external pressure. It is observed that as we increase the volume reinforcement of $SiC_{p}$, radial stresses in the cylinder becomes compressive in nature. It can be seen that when m=−0.5, radial stress has high compressiveness at outer radial points and decreases in magnitude at the outer boundary. Radial stress under m=0.5 gradation has lower magnitude as compared to the m=−0.5 case but otherwise has similar compressive behavior towards the outer radial points of the cylinder. When constant gradation m=0 is considered, the radial stress is tensile in nature for a larger part of the radius but becomes compressive at the outer radial points of the cylinder.

**Figure 26:**
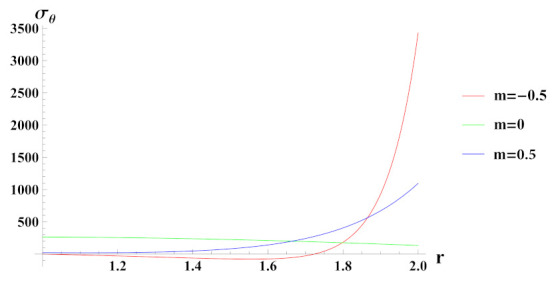
[PDM]Tangential stress (in MPa) along the radius *r* (in cm) of the cylinder, under the effect of rotation ($\omega^{2}$ = 50 rad/s), external pressure $p_{o}$ = 50 MPa and quadratic volume reinforcement.

Tangential stress in a rotating cylinder under external pressure is depicted in Figure 26. For m=0.5, it is tensile and increases from the inner to outer radius. When m=−0.5, tangential stress is found to be slightly compressive but become tensile towards the outer radius of cylinder. In a cylinder with constant gradation, the stress in the tangential direction decreases from the inner radius of the cylinder to its outer radius. It is to be noted that on increasing volume reinforcement gradation, tangential stresses are high at the outer radius as compared to the inner radial points.

**Figure 27:**
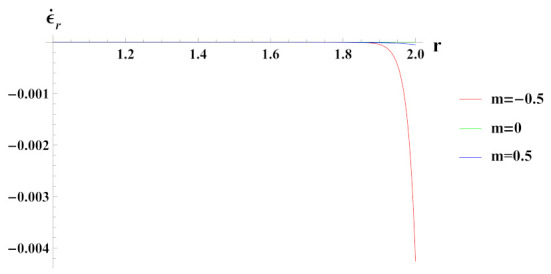
[PDM]Radial strain rate along the radius *r* (in cm) of the cylinder, under the effect of rotation ($\omega^{2}$ = 50 rad/s), external pressure $p_{o}$ = 50 MPa and quadratic volume reinforcement.

From Figure 27, concludes compressive nature of radial strain rate with the highest magnitude of compressiveness at the outer radius under for m=−0.5 and at inner radius for m=0, respectively.

**Figure 28:**
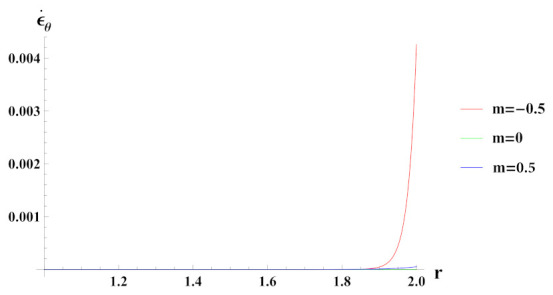
[PDM]Tangential strain rate along the radius *r* (in cm) of the cylinder, under the effect of rotation ($\omega^{2}$ = 50 rad/s), external pressure $p_{o}$ = 50 MPa and quadratic volume reinforcement.

Under external pressure, the tangential strain rate in cylinder as shown in Figure 28, is found to be high in magnitude at the outer radius, under m=−0.5 followed by m=0.5 and m=0 gradation cases.

## 4. Conclusions

This study investigated the performance of a rotating functionally graded cylinder in the presence of internal and external pressure. The impact of exponential, linear and quadratic volume reinforcement on secondary creep stresses–strains in a rotating cylinder has been analyzed. Some of the important findings from the study are reported below:

1. Radial stress in a rotating cylinder with an increasing exponential volume reinforcement gradation has higher magnitude and compressiveness as compared to increasing linear volume reinforcement gradation.
2. Radial stress in a rotating cylinder with decreasing exponential volume reinforcement gradation has lower magnitude as compared to decreasing linear volume reinforcement gradation.
3. Radial stresses in cylinder with quadratic volume reinforcement profile are found to be compressive throughout the radius. Its compressiveness increases towards the outer radial points of the cylinder as we increase the volume reinforcement. Further, the compressiveness at the outer radial points is higher in the case of internal pressure as compared to external pressure.
4. Tangential stresses in a rotating cylinder with increasing exponential volume reinforcement profile decreases from inner to outer radius of the cylinder whereas under increasing linear volume reinforcement profile it increases from inner to outer radius of the cylinder.
5. In a rotating cylinder with decreasing exponential volume reinforcement profile, tangential stress increases from inner to outer radius of the cylinder whereas under linear volume reinforcement profile, it decreases from inner to outer radius of the cylinder.
6. In case of quadratic volume reinforcement profile, tangential stress increase from inner to outer radius of the cylinder and their magnitude also increase with increase in volume reinforcement in cylinder.
7. Strain rates in radial and tangential directions of cylinder under decreasing exponential volume reinforcement profile are of higher magnitude under internal pressure as compared to external pressure. In the case of linear volume reinforcement, it has higher magnitude under internal pressure as compared to external pressure. Further, under decreasing exponential volume reinforcement profile, their magnitude increases from the inner to outer radius whereas in the linear volume reinforcement profile, their magnitude decreases from the inner to outer radius.
8. Strain rates in radial and tangential directions of a cylinder with quadratic reinforcement are higher in magnitude under internal pressure as compared to external pressure. Further, with an increase in volume reinforcement, their magnitude increases from the inner to outer radius of the cylinder.
9. Thus, based on the obtained results, a comparison of creep stresses and strain rates among rotating cylinders with linearly and non-linearly varying volume reinforcement profile is presented. It can be observed from the above study outcomes that the nature of reinforcement function of $SiC_{p}$ in Al metal matrix at inner and outer radial surfaces causes a significant effect on the magnitude of creep stresses and strain rates at inner and outer radial surfaces, under internal/external pressure conditions.

## Data Availability

Not applicable.

## Abbreviations

The following abbreviations are used in this manuscript:

FGM	Functionally Graded Material
SiCp	Silicon Carbide Particulates

## Appendix A

Substituting Equation (17) into Equation (11), we obtain

$$\frac{d\sigma_{r}}{dr}=\frac{I_{1}}{r^{\frac{n+2}{n}}}-\left(\rho_{m}+\frac{\left(\rho_{c}-\rho_{m}\right)}{100}e^{-n_{1}{\left(\frac{r}{r_{o}}\right)}^{2}}\right)\omega^{2}r$$

On integrating between $r_{i}$ to *r*, we obtain

$$\int_{r_{i}}^{r}\frac{d\sigma_{r}}{dr}=\int_{r_{i}}^{r}\frac{I_{1}}{r^{\frac{n+2}{n}}}dr-\int_{r_{i}}^{r}\left(\rho_{m}+\frac{\left(\rho_{c}-\rho_{m}\right)}{100}e^{-n_{1}{\left(\frac{r}{r_{o}}\right)}^{2}}\right)\omega^{2}rdr$$

Using the boundary condition at $r=r_{i}$, we have ${\left(\sigma_{r}\right)}_{r=r_{i}}=-p_{i}$ and hence the above equation reduces as given below:

$$\sigma_{r}-{\left(\sigma_{r}\right)}_{r=r_{i}}=\int_{r_{i}}^{r}\frac{I_{1}}{r^{\frac{n+2}{n}}}dr-\frac{\rho_{m}\omega^{2}\left(r^{2}-{r_{i}}^{2}\right)}{2}+\frac{\left(\rho_{c}-\rho_{m}\right){r_{o}}^{2}\omega^{2}}{\left(100\right)\left(2n_{1}\right)}\left[e^{-n_{1}\left(\frac{r^{2}}{{r_{o}}^{2}}\right)}-e^{-n_{1}\left(\frac{{r_{i}}^{2}}{{r_{o}}^{2}}\right)}\right]$$

(A1) $$\sigma_{r}=\int_{r_{i}}^{r}\frac{I_{1}}{r^{\frac{n+2}{n}}}dr-\frac{\rho_{m}\omega^{2}\left(r^{2}-{r_{i}}^{2}\right)}{2}+\frac{\left(\rho_{c}-\rho_{m}\right)\omega^{2}}{100}\frac{{r_{o}}^{2}}{2n_{1}}\left[e^{-n_{1}\left(\frac{r^{2}}{{r_{o}}^{2}}\right)}-e^{-n_{1}\left(\frac{{r_{i}}^{2}}{{r_{o}}^{2}}\right)}\right]-p_{i}$$

## References

[1] You L.H., Ou H., Zheng Z.Y. (2007). Creep deformations and stresses in thick-walled cylindrical vessels of functionally graded materials subjected to internal pressure. Compos. Struct..

[2] Chen J.J., Tu S.T., Xuan F.Z., Wang Z.D. (2007). Creep analysis for a functionally graded cylinder subjected to internal and external pressure. J. Strain Anal. Eng. Des..

[3] Sharma S., Sahni M., Kumar R. (2009). Elastic-plastic transition of transversely isotropic thick-walled rotating cylinder under internal pressure. Def. Sci. J..

[4] Atabakhshian V., Loghman A. (2012). Semi-analytical solution for time-dependent creep analysis of rotating cylinders made of anisotropic exponentially graded material (EGM). J. Solid Mech..

[5] Nejad M.Z., Abedi M., Lotfian M.H., Ghannad M. (2012). An exact solution for stresses and displacements of pressurized FGM thick-walled spherical shells with exponential-varying properties. J. Mech. Sci. Technol..

[6] Sharma S., Aggarwal A.K., Sharma R. (2013). Safety analysis of thermal creep non-homogeneous thick-walled circular cylinder under internal and external pressure using Lebesgue strain measure. Multidiscip. Model. Mater. Struct..

[7] Sahni M., Sharma S. Analysis of safety measure in creep transversely isotropic thick-walled rotating cylinder by finitesimal deformation under external pressure. Proceedings of the 3rd International Conference on Reliability, Infocom Technologies and Optimization (ICRITO).

[8] Kashkoli M.D., Nejad M.Z. (2014). Effect of heat flux on creep stresses of thick-walled cylindrical pressure vessels. J. Appl. Res. Technol..

[9] Vedeld K., Sollund H.A., Hellesland J. (2015). Closed analytical expressions for stress distributions in two-layer cylinders and their application to offshore lined and clad pipes. J. Offshore Mech. Arct. Eng..

[10] Loghman A., Shayestemoghadam H. (2016). Magneto-thermo-mechanical creep behavior of nano-composite rotating cylinder made of polypropylene reinforced by MWCNTs. J. Theor. Appl. Mech..

[11] Kalai A.T., Moud S.H., Hassani B. (2016). Elasto-plastic stress analysis in rotating disks and pressure vessels made of functionally graded materials. Lat. Am. J. Solids Struct..

[12] Sharma S., Yadav S. Thermo creep analysis of thick-walled functionally graded cylinder under internal and external pressure. Proceedings of the AIP Conference Proceedings, Mathematical Sciences and its Applications.

[13] Celebi K., Yarimpabu D., Keles I. (2017). A novel approach to thermal and mechanical stresses in a FGM cylinder with exponentially-varying properties. J. Theor. Appl. Mech..

[14] Bakhshizadeh A., Nejad M., Kashkoli M.D. (2017). Time-dependent hygro-thermal creep analysis of pressurized FGM rotating thick cylindrical shells subjected to uniform magnetic field. J. Solid Mech.1.

[15] Sahni M., Sahni R., Mehta P. Creep Behaviour under SiCp Exponential Volume Reinforcement in FGM Composite Rotating Cylinders. Proceedings of the International Conference on Recent Trends in Engineering and Material Sciences (ICEMS-2016).

[16] Habib E.S., El-Hadak M.A., El-Megharbel A. (2018). Stress analysis for cylinder made of FGM and subjected to thermo-mechanical loadings. Metals.

[17] Kashkoli M.D., Tahan K.N., Nejad M.Z. (2018). Thermo-mechanical creep analysis of FGM thick cylindrical pressure vessels with variable thickness. Int. J. Appl. Mech..

[18] Çallioğlu H., Sayer M., Demir E. (2015). Elastic-plastic stress analysis of rotating functionally graded discs. Thin-Walled Struct..

[19] Mehta P.D., Sahni M. (2020). Thermo-Mechanical Analysis for an Axisymmetric Functionally Graded Rotating Disc under Linear and Quadratic Thermal Loading. Int. J. Math. Eng. Manag. Sci..

[20] Mehta P.D., Mishra L., Sahni M. (2019). Thermomechanical Stress Analysis of Thick-Walled Cylinder with Inner FGM Layer. Struct. Integr. Life.

[21] Hajisadeghian A., Masoumi A., Parvizi A. (2018). Investigating the magnetic field effects on thermomechanical stress behavior of thick-walled cylinder with inner FGM layer. J. Therm. Stresses.

[22] Dini A., Nematollahi M.A., Hosseini M. (2021). Analytical solution for magneto-thermo-elastic responses of an annular functionally graded sandwich disk by considering internal heat generation and convective boundary condition. J. Sandw. Struct. Mater.

[23] Singh T., Gupta V.K. (2010). Modeling steady state creep in functionally graded thick cylinder subjected to internal pressure. J. Compos. Mater.

[24] Garg M., Deepak D., Gupta V.K. (2014). FE modeling of creep in linear and non-linear FGM cylinder under internal pressure. Multidiscip. Model. Mater. Struct..

[25] Aggarwal A.K., Sharma R., Sharma S. (2014). Collapse pressure analysis of transversely isotropic thick-walled cylinder using Lebesgue strain measure and transition theory. Sci. World J..

[26] Nie G.J., Batra R.C. (2010). Material tailoring and analysis of functionally graded isotropic and incompressible linear elastic hollow cylinders. Compos. Struct..

[27] Murata A., Karwowski W. (2021). On the Root Causes of the Fukushima Daiichi Disaster from the Perspective of High Complexity and Tight Coupling in Large-Scale Systems. Symmetry.

[28] Mao J.F., Zhu J.W., Bao S.Y., Luo L.J., Gao Z.L. (2015). Creep and damage analysis of reactor pressure vessel considering core meltdown Scenario. Procedia Eng..

